# Concordance of anaplastic lymphoma kinase (*ALK*) gene rearrangements between circulating tumor cells and tumor in non-small cell lung cancer

**DOI:** 10.18632/oncotarget.8136

**Published:** 2016-03-16

**Authors:** Chye Ling Tan, Tse Hui Lim, Tony KH Lim, Daniel Shao-Weng Tan, Yong Wei Chua, Mei Kim Ang, Brendan Pang, Chwee Teck Lim, Angela Takano, Alvin Soon-Tiong Lim, Man Chun Leong, Wan-Teck Lim

**Affiliations:** ^1^ Department of Pathology, Singapore General Hospital, Singapore; ^2^ Department of Medical Oncology, National Cancer Center Singapore, Singapore; ^3^ Department of Molecular Oncology, National University Health System Singapore, Singapore; ^4^ Faculty of Engineering, Department of Biomedical Engineering, National University of Singapore, Singapore; ^5^ Mechanobiology Institute, National University of Singapore, Singapore; ^6^ Clearbridge Biomedics Pte Ltd, Singapore; ^7^ Institute of Molecular and Cell Biology, Singapore

**Keywords:** ALK-gene rearrangement, circulating tumor cells, fluorescent in-situ hybridization, lung cancer, molecular diagnosis

## Abstract

Anaplastic lymphoma kinase (*ALK)* gene rearrangement in non-small cell lung cancer (NSCLC) is routinely evaluated by fluorescent in-situ hybridization (FISH) testing on biopsy tissues. Testing can be challenging however, when suitable tissue samples are unavailable. We examined the relevance of circulating tumor cells (CTC) as a surrogate for biopsy-based FISH testing. We assessed paired tumor and CTC samples from patients with *ALK* rearranged lung cancer (*n* = 14), *ALK*-negative lung cancer (*n* = 12), and healthy controls (*n* = 5) to derive discriminant CTC counts, and to compare *ALK* rearrangement patterns. Blood samples were enriched for CTCs to be used for *ALK* FISH testing. *ALK*-positive CTCs counts were higher in *ALK*-positive NSCLC patients (3–15 cells/1.88 mL of blood) compared with *ALK*-negative NSCLC patients and healthy donors (0–2 cells/1.88 mL of blood). The latter range was validated as the ‘false positive’ cutoff for *ALK* FISH testing of CTCs. *ALK* FISH signal patterns observed on tumor biopsies were recapitulated in CTCs in all cases. Sequential CTC counts in an index case of lung cancer with no evaluable tumor tissue treated with crizotinib showed six, three and eleven *ALK*-positive CTCs per 1.88 mL blood at baseline, partial response and post-progression time points, respectively. Furthermore, *ALK* FISH rearrangement suggestive of gene copy number increase was observed in CTCs following progression. Recapitulation of *ALK* rearrangement patterns in the tumor on CTCs, suggested that CTCs might be used to complement tissue-based *ALK* testing in NSCLC to guide *ALK*-targeted therapy when suitable tissue biopsy samples are unavailable for testing.

## INTRODUCTION

Lung cancer accounts for about 13% of all cancer diagnoses and remains the leading cause of death by cancer in the world [1], with almost 70% of patients diagnosed with locally advanced or metastatic disease at presentation [1, 2]. Non-small cell lung cancer (NSCLC) accounts for approximately 85% of all cases of lung cancer and is associated with poor prognosis [2]. The 5-year overall survival rate for NSCLC across all stages is only 21% and is even lower (~5%) for stages IIIB and IV [1, 3].

Oncogenic ‘driver mutations’ have now been identified in various subsets of NSCLC [4-6]. Of these drivers, somatic mutations in epidermal growth factor receptor (EGFR) and anaplastic lymphoma kinase (*ALK)* [5-7] are the most frequently described. In 2007, researchers identified the presence of a chimeric ALK protein with fibroblast-transforming properties that was formed following fusion of the echinoderm microtubule-associated protein-like 4 (*EML4*) and *ALK* genes [6]. *EML4*-*ALK* subverts intracellular signaling pathways to promote tumor cell survival and growth [8]. The overall incidence of *ALK* gene rearrangement in NSCLC ranges between 0.4% and 13.4%, and is similar in both Asian and Western populations [9]. This discovery resulted in the accelerated development and approval by the U.S. Food and Drug Administration (FDA) of the *ALK*-targeting tyrosine kinase inhibitors (TKIs) crizotinib (Xalkori^®^, Pfizer, New York, USA) in 2011, and ceritinib (Zykadia™, Novartis, Basel, Switzerland) in 2014 to treat patients with metastatic NSCLC who express the abnormal *ALK* gene [10, 11].

The true therapeutic benefit of novel molecules targeting the mutant ALK fusion protein in NSCLC relies on identifying the right patient population for treatment, and on detecting the emergence of tumor resistance. The American Society of Clinical Oncology (ASCO) endorsed the joint College of American Pathologists (CAP)/International Association for the Study of Lung Cancer (IASLC)/Association for Molecular Pathology (AMP) clinical practice guideline on EGFR and *ALK* molecular testing for patients with lung cancer, which holds that an *ALK* fluorescent *in-situ* hybridization (FISH) assay using dual-labeled break-apart probes is the preferred testing methodology to detect *ALK* gene rearrangement [12].

The accuracy of testing nonetheless depends on the quality of tumor biopsies. Approximately 50% of NSCLC patients who undergo re-biopsy for determination of resistance after first-line chemotherapy have insufficient/non-diagnostic biopsy specimens or cytology samples available for molecular testing [13]. Biopsy is invasive and repeating the procedure is not always feasible due to safety concerns and general unwillingness of patients, among other reasons [14]. Furthermore, lung adenocarcinomas are heterogeneous with a diverse and ever-evolving genetic and epigenetic makeup that contributes towards treatment resistance [15]. These barriers to biopsy collectively pose a challenge to track oncogene activity in real-time over the course of treatment. There is a need for a minimally invasive assay for tumor molecular profiling and continuous treatment monitoring in order to provide timely and tailored cancer treatment.

Circulating tumor cells (CTCs) released from the primary tumor site into the circulation represent a potential means of non-invasively isolating tumor cells for *ALK* FISH testing and other molecular characterizations. Recent data supports the role of these renegade cells as seeds of cancer metastases [16, 17]. They may recapitulate the phenotypic heterogeneity and molecular signatures of the primary tumor, as well as that of metastatic lesions [18-20]. While their presence and prevalence in blood are often associated with poor prognosis [21], CTCs may hold further relevance as an alternative tumor source, which can complement existing tissue-based diagnostic tests, especially when biopsy material is absent or inadequate. In patients with lung adenocarcinoma, hypothesis-generating studies have strongly suggested that *ALK* status could be determined based on testing of CTCs, with comparable results as testing of tumor tissues [22, 23].

The key challenge of CTC-based testing is the enrichment and isolation of these cells within an acceptable timeframe. Various technical approaches have been used to isolate these CTCs [19, 24, 25]. They can be broadly categorized into antibody affinity-based, imaging-based and size-based techniques [26]. The only current US FDA-approved CTC capturing technology utilizes EpCAM immunomagnetic means to isolate EpCAM-positive CTCs for prognostic purposes and would inadvertently miss out on EpCAM-negative CTCs [27, 28]. As a predictive biomarker for treatment monitoring and molecular analysis, it is pertinent to ensure reliable and reproducible isolation of CTCs of different phenotypic and molecular subtypes for various pre-and post-treated patient cohorts [25]. An increasing body of evidence suggests that non-immunomagnetic-based CTC technologies can reliably retrieve a comprehensive population of CTCs for molecular subtyping [19, 28].

Here, we evaluated the feasibility of an antibody-independent CTC isolation system using lung adenocarcinomas that have been tested *ALK* positive as a model to examine the concordance patterns between CTCs and tumor tissue, and to determine whether CTCs were reproducibly detectable in circulation. We further explored the potential use of CTCs in lung cancer, as a surrogate for molecular testing of the primary tumor for *ALK* gene rearrangement.

## RESULTS

### Study group

We prospectively recruited 27, mostly late-stage NSCLC patients, 14 of whom had *ALK-*rearranged and 12 had wild-type *ALK*, determined from the initial biopsy diagnoses. One patient in the cohort, who was not from Singapore, had an unknown *ALK* status due to incomplete referral records. Sixty percent of patients were males. All *ALK*-positive patients were non-smokers. Five healthy donors (three males and two females), aged between 18-55 years old with no history of cancer, were also recruited into the control cohort. As *ALK* translocation in NSCLC patients is strongly correlated with a non-smoker or light smoker status [29], these healthy donors were non-smokers. The clinicopathologic features of the study group are summarized in Table 1.

**Table 1:** Clinicopathological characteristics of patients enrolled in this study

Patient characteristics	Cases (%) *N* = 27
Age, years	32–76
Sex	
Male	16 (59.3%)
Female	11 (40.7%)
Smoking history	
Non-smoker	16 (59.3%)
Smoker	5 (18.5%)
Ex-smoker	5 (18.5%)
No info	1 (3.7%)
Clinical staging	
IB	1 (3.7%)
IIIA	1 (3.7%)
IIIB	2 (7.4%)
IV	23 (85.2%)
Histological subtype	
ALK-positive	14 (51.9%)
Adenocarcinoma (NSCLC)	11 (40.7%)
Unknown subtype (NSCLC)	3 (11.1%)
ALK-negative	12 (44.4%)
Adenocarcinoma (NSCLC)	5 (18.5%)
Unknown subtype (NSCLC)	7 (25.9%)
ALK status unknown	1 (3.7%)
Adenocarcinoma (NSCLC)	1 (3.7%)

Abbreviations: ALK, anaplastic lymphoma kinase; NSCLC, non-small cell lung cancer

### Histopathological analysis of tumor tissues

**Figure 1:**
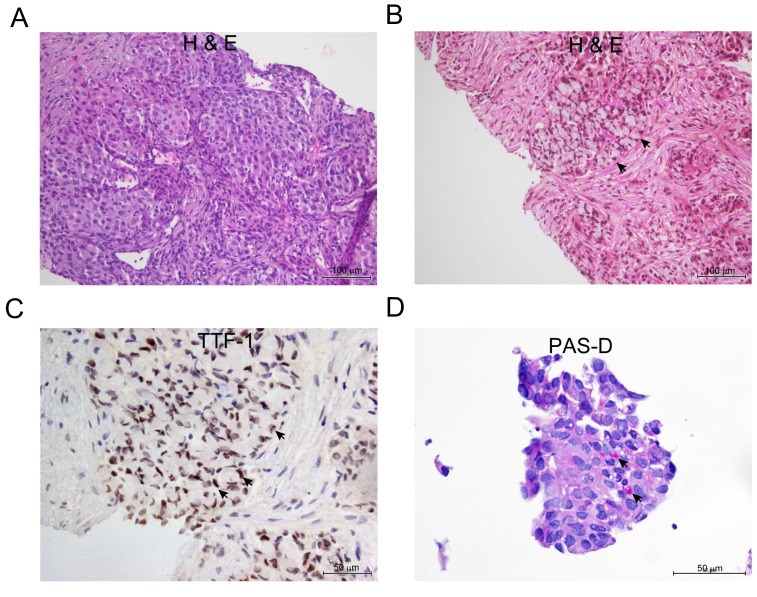
Representative appearance of NSCLC adenocarcinoma with signet ring cells features **A.** H&E stain showing solid nests of tumor cells **B.** Solid with signet ring cells (arrow ) **C.** Thyroid transcription factor-1 (TTF-1) IHC stain showing strong nuclear reaction in the signet ring cells. **D.** Solid tumor showing focal positive reaction for (PAS-D) within mucin vacuoles (arrow).

In the *ALK*-positive group, three out of 14 tumor tissues exhibited morphology that is associated with *ALK* rearrangements [30]. Histological preparations showed solid adenocarcinoma with signet ring cells (Figure 1A and 1B), and cribrifom adenocarcinoma with focal squamoid cells (not shown). Immunohistochemical (IHC) studies showed strong and diffuse nuclear reaction for Thyroid Transcription Factor-1 (TTF-1). This finding confirmed the diagnosis of adenocarcinoma of lung origin in this particular setting (Figure 1C). Some tumors also showed focal reaction to periodic acid-Schiff with diastase (PAS-D) within mucin vacuoles, which is a general feature of adenocarcinomas, as opposed to squamous cell carcinomas (Figure 1D) [30].

### Concordance in *ALK* rearrangement pattern between CTCs and tumor

**Figure 2:**
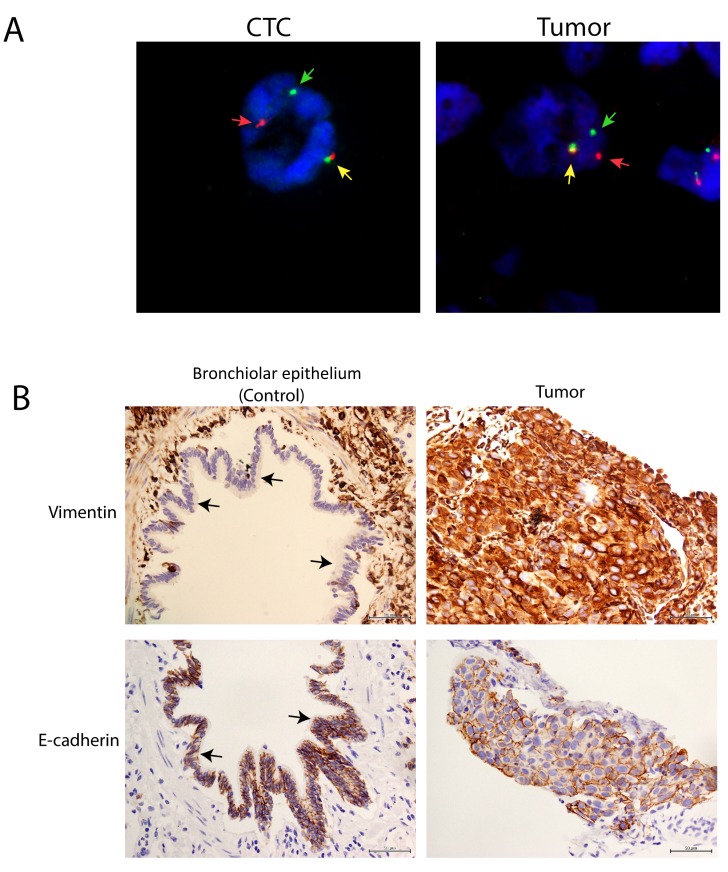
High concordance of *ALK* FISH rearrangements patterns between CTCs and tumors in NSCLC adenocarcinoma patients **A**. Representative *ALK* FISH rearrangement patterns in CTCs and tumors showing 1F1R1G rearrangement patterns. Yellow, red and green arrows represent fusion (F), orange (R) and green (G) fluorescent signals. **B**. Representative vimentin (upper panel) and E-cadherin (lower panel) IHC in tumor and control bronchiolar epithelium (black arrow).

Following FISH testing on all tumor samples in the cohort, it was found that *ALK*-positive tumors harbored *ALK* rearrangements with various patterns of abnormality (Table 2). The majority of the tumor samples harbored the one fusion (F) and one split orange (R) and green (G) signal (Figure 2A). The tumor from Patient P5 presented various *ALK* rearrangement patterns such as 1F1R1G, 2F1R, 2F2R, 1F1R and 1F2R.

FISH testing was subsequently performed on CTCs that were enriched and isolated from the matched blood samples. Data showed that *ALK* rearrangement patterns (majority 1F1R1G) observed in primary tumor tissues were recapitulated on most of the *ALK*-positive CTCs, giving an overall concordance rate of over 90% based on the 1F1R1G fusion pattern (Table 2). In Patient P5 (Table 2), the CTCs were able to recapitulate three out of five *ALK* rearrangement patterns observed in the tumor tissue.

**Table 2:** Concordance of *ALK* rearrangement patterns between CTC and tumor in patients with *ALK*-positive NSCLC

Case number of patients with *ALK*-positive NSCLC	*ALK* rearranged/total cells scored (Tumor)	*ALK* rearrangement patterns (% of tumor cells observed with respective patterns)	
		Tumor	CTC
P1	61/100	1F1R1G (100%)	1F1R1G (100%)
P2	45/100	1F1R1G (100%)	1F1R1G (100%)
P3	79/100	1F1R1G (100%)	1F1R1G (100%)
P4	30/100	1F1R1G (100%)	1F1R1G (100%)
P5	61/100	1F1R1G (4.9%) 2F1R (34.4%) 2F2R (3.3%) 1F1R (49.2%) 1F2R (8.2%)	1F1R1G (50%) 2F1R (37.5%) 2F2R (12.5%)
P6	72/100	1F1R1G (29.2%) 1F1R (70.8%)	1F1R1G (100%)
P7	81/100	1F1R (100%)	1F1R (75%) 1F1R1G (25%)
P8	55/100	1F1R1G (100%)	1F1R1G (100%)
P9	77/100	1F1R (31.2%) 1F1R1G (68.8%)	1F1R (100%)
P10	100/100	1F1R (100%)	1F1R (50%) 1F1R1G (50%)
P11	62/100	1F1R1G (100%)	1F1R1G (100%)
P12	77/100	1F1R1G (100%)	1F1R1G (100%)
P13	45/100	1F1R1G (100%)	1F1R1G (100%)
P14	Not available	Not available	1F1R1G (100%)

Abbreviations: ALK, anaplastic lymphoma kinase; CTC, circulating tumor cells; NSCLC, non-small cell lung cancer.

We further observed an overexpression of vimentin in the tumor samples, along with the control bronchiolar epithelium (Figure 2B). However, loss of E-cadherin was not obvious in these samples.

**Figure 3:**
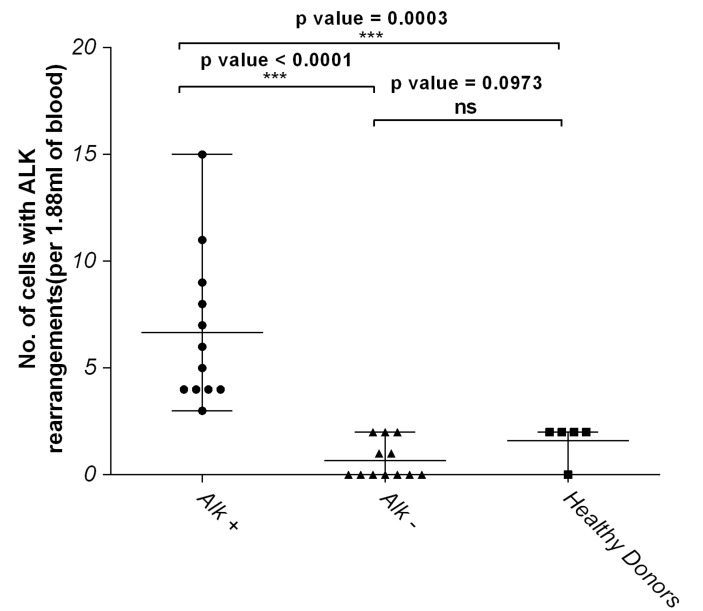
Number of cells with *ALK* rearrangements in *ALK*-positive NSCLC patients is significantly higher compared to *ALK*-negative and healthy donors Graph represents statistical analyses of the data on Table S1 using the non-parametric two-tailed *t*-test. NS represents not significant while *p* value <0.05 were considered significant.

The number of *ALK*-positive rearranged CTCs retrieved from *ALK*-positive patients was significantly enriched compared with *ALK*-negative patients (*p* < 0.0001) and healthy donors (*p* = 0.0003) (Figure 3).

### Establishment and validation of *ALK* break-apart probes cutoff in *ALK*-negative samples

*ALK* testing by FISH in NSCLC tumor tissues without *ALK* rearrangement may detect rearrangement-positive patterns (i.e. split patterns or isolated 3’ patterns) in a fraction of cells [31-33], likely because of truncation artefact caused by tissue sectioning, or perhaps a stochastic genomic alteration that does not indicate a specific gene fusion. *ALK* FISH testing in formalin-fixed, paraffin-embedded (FFPE) NSCLC tumor tissues has a ‘false positive’ cutoff value of 15% to allow for the best separation between *ALK*-rearranged and *ALK* wild-type cells [31, 33]. However, it is not possible to apply this guideline in the *ALK* FISH testing on CTCs because the number of CTCs in any given blood sample would be too low.

In our study, we established and validated the ‘false positive’ cutoff for *ALK* FISH in CTCs using 12 blood samples from NSCLC *ALK*-negative patients and five blood samples from healthy donors (Supplementary Data). Results from the *ALK*-negative NSCLC cohort scored a median of two or less positive cells (range 0-2 cells/1.88 mL blood). The result concurred with the numbers observed for healthy blood samples. In fact, no statistical difference in *ALK*-positive cell counts was observed between *ALK*-negative NSCLC cohort and healthy donors (*p* = 0.0973) (Figure 3). This data established the ‘false-positive’ cutoff for *ALK* break-apart probes in CTCs at ≤ two cells per 1.88 mL blood.

### Potential clinical applications

Sequential CTC enumeration and FISH was performed on blood samples from a patient with no accessible tissue for *ALK* FISH testing. The index case was a never smoker male diagnosed with NSCLC. A transthoracic needle aspiration biopsy was performed on the right hilar mass to obtain a specimen for histological analysis. The hematoxylin and eosin (H&E) stain showed one small cluster of NSCLC cells with strong nuclear reaction for TTF-1 favoring adenocarcinoma. Unfortunately, his diagnostic tissue was exhausted and no further molecular profiling could be performed. He did not respond to EGFR tyrosine kinase inhibitor (TKI) therapy and had a short duration of response to pemetrexed and cisplatin. Re-biopsy of the lung and liver tumors was considered but the patient declined due to the risk of bleeding. He consented to blood sampling instead; the sample was subsequently processed as described in the Methods section.

**Figure 4:**
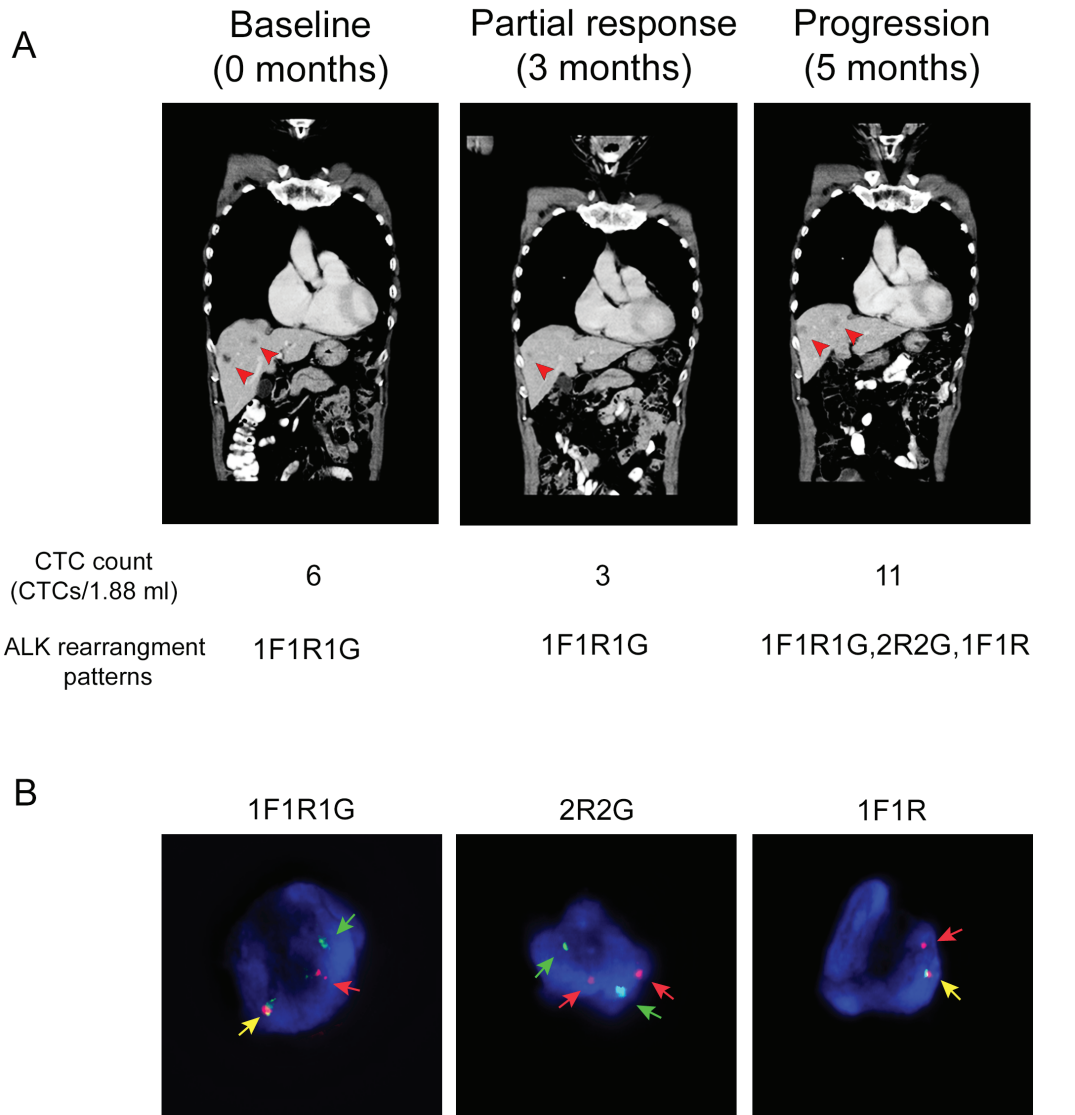
An index case suggests that ALK-rearranged CTCs could have clinical application as a diagnostic biomarker to monitor crizotinib treatment and response **A.** CT scan taken at baseline, partial response and progression time points showing presence of metastatic tumor in liver (arrow). CTC counts and *ALK* rearrangement patterns for each time point is indicated in the lower panel. **B.** Representative images showing 1F1R1G, 2R2G and 1F1R *ALK* rearrangement patterns following progression on crizotinib treatment. Yellow, red and green arrows represent fusion (F), orange (R) and green (G) fluorescent signals.

At baseline, six CTCs displaying a 1F1R1G pattern were isolated and met the necessary cutoffs for *ALK*-positivity (Figure 4A). A trial of crizotinib was commenced. Confirmatory scans done 3 months after completion of treatment demonstrated good partial response in the liver and minor response in the primary lung tumor, based on RECIST criteria (Figure 4A). Sequential CTC counts dropped to three cells displaying the similar *ALK* rearranged pattern as the baseline. He continued on crizotinib but unfortunately, his disease progressed in the liver and the brain 5 months after treatment initiation (Figure 4A). A post-progression blood sample showed additional *ALK* rearrangement patterns present in his CTCs, which differed from the baseline patterns. New *ALK* rearrangement patterns such as 2R2G and 1F1R appeared, in addition to 1F1R1G, which was previously present (Figure 4B). The number of *ALK*-positive CTCs also increased from three to eleven CTCs per 1.88 mL of blood post-progression.

## DISCUSSION

We successfully captured CTCs using an antibody-independent CTC isolation system. CTCs were enriched from the blood samples collected from 27 NSCLC patients, 14 of whom were *ALK*-positive. Three of the cases exhibited solid with signet ring cells pattern associated with *ALK* positivity [34-37]. Overall, CTCs isolated from the *ALK*-positive patient cohort were above detectable levels, even among previously treated patients.

The presence of *ALK* rearrangement in CTCs was previously analyzed and reported by French groups using the Isolation by Size of Epithelial Tumor (ISET) system, which is also an antibody-free system (Rarecells, Paris, France) [22, 23]. They characterized 18 *ALK*-positive lung tumor and CTC samples showing high concordance in *ALK* rearrangement among European patient cohort. In agreement with our data reported in this study, ‘false-positive’ signals were similarly observed on CTCs from *ALK*-negative samples and a 4 cell/mL cutoff was eventually established [22, 38].

In a similarly designed study, Pailler et al. [22] described high concordance in *ALK* rearrangement patterns between the CTC and tumor biopsies in 18 *ALK*-positive and 14 *ALK*-negative patients with metastatic NSCLC. This percentage of concordance is in agreement with our own results. They reported that all *ALK*-positive NSCLC patients in their cohort had 4 or more *ALK*-rearranged CTCs per mL of blood. The study did not include healthy donors to establish a ‘false positive’ cutoff for *ALK* FISH testing of CTCs. They further reported that CTCs harboring the 1F1R1G *ALK* rearrangement patterns is associated with epithelial-mesenchymal transition (EMT) phenotype [22].

Another study had reported that the EMT phenotype (represented by loss of E-cadherin and expression of vimentin) was more common in *ALK*-rearranged tumors than other genotypes (38.9%, 19.1%, 26.9% and 14.6% of *ALK*-rearranged, EGFR-mutated, K-ras mutated and triple negative tumors, respectively; *p* = 0.015) [39]. Separately, expression of vimentin alone was detected in 49.30% of *ALK*-rearranged tumors while loss of E-cadherin was detected in 71.30% [39]. In our study, we also observed an overexpression of vimentin in the tumor samples in comparison with the control bronchiolar epithelial tissue. However, the loss of E-cadherin was not obvious in our tumor samples, which suggested that some of the tumor cells retained their epithelial characteristics within a heterogeneous population of cells. The predominance of this particular *ALK* rearrangement pattern in our patient's CTCs is therefore consistent with the observation above suggesting that these tumors and their CTCs may be favoring the EMT pathway.

This study further highlights the utility of antibody-independent microfluidic isolation systems for the isolation and downstream characterization of CTCs compared with immunomagnetic antibody-dependent systems. While the numbers of CTCs isolated here are small and may present substrate limitations to downstream characterization of CTC, it should be noted that the current numbers were derived from <2 mL of blood, as opposed to existing systems which use up to 7.5 mL of blood or more. In addition, we have previously demonstrated that there is an association between CTC number and the volume of blood processed [19]. This suggests a limitation that can be easily overcome.

The index case presented here raises the possibility that CTC enumeration based on *ALK* FISH may be associated with treatment response with crizotinib by imaging. The appearance of additional *ALK* rearrangement patterns following progression with crizotinib treatment exhibited a double split in both *ALK* alleles giving rise to the 2R2G *ALK* rearrangement pattern. The additional copy of the oncogenic *ALK* may have contributed to disease progression despite treatment with an *ALK* inhibitor. This observation is worthy of further inquiry, because while the presence of *ALK* copy number gain is correlated with crizotinib resistance, as previously reported by Doebele *et al*. [40], and *in vitro* studies have identified potential resistance mutations in the *ALK* gene, for example L1196M, G1269A, S1206Y and G1202R [40, 41], limited analysis of post-progression biopsies of tumors from a phase 1 study of LDK378 suggested that these secondary resistance mutations or gene amplification do not account for a majority of resistance cases [11]. Hence, further work with paired re-biopsies and sequential CTC collection may assist understanding of resistance mechanisms in *ALK*-driven tumors.

Conclusions drawn from our study are limited by its relative small patient population, as was Pallier's study [22]. Nonetheless, the converging trend of both studies’ findings is indicative of the utility and potential of CTCs as an alternate target of *ALK* testing in lung cancer and informs the development of CTC-based technology. More importantly, these studies provide the basis for subsequent, large-scale validation studies.

In summary, high concordance of *ALK* rearrangement patterns in CTCs and tumors as assessed by *ALK* FISH testing indicates that CTCs may have utility as a non-invasive surrogate diagnostic tool and may be useful in the longitudinal follow-up for resistance profiling. The availability of a non-invasive tool would improve efforts to guide *ALK*-rearranged targeted treatment in NSCLC, especially in cases without tissue availability. Further efforts at downstream CTC characterization and culture following enrichment are ongoing.

## MATERIALS AND METHODS

### Patient recruitment and blood samples

Patients with confirmed NSCLC were recruited into this trial. They were naïve for *ALK*-targeted TKI treatment, but may have received other forms of chemotherapy. Once informed consent was secured from these patients, their blood samples were processed for CTC analysis. The clinical sample collection protocols were reviewed and approved by SingHealth Centralised Institutional Review Board. Clinicopathological information was also recorded for these patients. Blood samples from healthy donors were used as controls in this study.

### Histopathology

H&E was done using the Leica ST5010 XL automated stainer (Leica Biosystems, Wetzlar, Germany) while periodic PAS-D staining was done using the Ventana BenchMark Special Stains automated stainer (Ventana Medical Systems Inc, Tucson, Arizona, USA), following the respective standard protocols. Histological diagnoses were made based on the World Health Organization (WHO) classification [42]. IHC labeling was performed on the Ventana BenchMark Ultra autostainer (Ventana Medical Systems Inc, Tucson, Arizona, USA) using the UltraView detection kit and proprietary Standard CC1 (SC1) pre-treatment sets. The antibodies used with their dilution and pre-treatments were as follows: TTF-1 (Novacastra NCL-TTF-1, clone SPT24, SC1, dilution 1:30), vimentin (DAKO M0725, clone V9, SC1, 1:100) and E-cadherin (Dako M3612, clone MCH-38, SC1, 1:30) antibodies. Histopathology data was reviewed by pathologists who had been accredited by the College of American Pathologists (CAP).

### CTC enrichment

Peripheral blood was collected using K2 EDTA vacutainer^®^ blood collection tube (BD, Singapore) and processed within 24 hours. Subsequently, 7.5 mL of whole blood was incubated with red blood cell (RBC) lysis buffer (G-Biosciences, USA) according to manufacturer's recommendations. Lysed RBCs in the supernatant were discarded after centrifugation. Remaining cell pellet containing CTCs was resuspended in ClearCell^®^ resuspension buffer prior to CTC enrichment using the ClearCell^®^ FX system (Clearbridge BioMedics, Singapore), according to manufacturer's instruction.

The ClearCell^®^ FX system is an automated CTC enrichment system driven by the CTChip^®^ FR1, a microfluidic biochip to isolate CTCs based on size, deformability and inertia. The isolation principle takes advantage of the inherent Dean vortex flows present in curvilinear channels for CTC enrichment, termed Dean Flow Fractionation (DFF) [43]. The enriched CTC sample output was equally divided into four portions.

### Fluorescent *in-situ* hybridization

Four μm thick FFPE tumor tissue sections were mounted on positively charged slides and deparaffinized.

FISH was subsequently performed using the US FDA-approved Vysis *ALK* Break Apart FISH Probe Kit (Abbott Molecular, Abbott Park, Des Plaines, IL, USA). The 5’ *ALK* probe was labeled with SpectrumGreen™ (G) and the 3’ *ALK* probe with SpectrumOrange™ (R). *ALK* FISH for FFPE tissues were considered positive if at least 15 % of the tumor cells showed abnormal break apart signals as detailed in the IVD Vysis *ALK* Break Apart FISH Probe Kit and by Camidge *et al.* [33]. A cell is interpreted as having a split pattern (*ALK*-positive) when the 5’ (G) and 3’ (R) signals are separated by two or more signal diameters. Cells lacking both fluorescent signals were not evaluated.

One portion (one-quarter) of the enriched CTCs was fixed in Shandon CytoSpin^TM^ Collection Fluid (ThermoFisher Scientific, USA) overnight at 4°C. The sample was deposited onto positively charged glass slides by cytospin (800 rpm, 5 mins). All the cells on the slides were analyzed for the *ALK* break-apart signal at 1000X magnification. The scorers analyzing the *ALK* break-apart signal on CTCs were blinded to the *ALK* rearrangement patterns on the tumor samples, as well as whether the cell isolated was from patients or healthy controls.

The remaining three portions of the enriched CTCs were stored at 4°C under validated conditions for future molecular testing.

### Data analysis

Statistical analyses of the data were performed in GraphPad Prism version 5.00 (GraphPad Software, San Diego, CA, USA). A non-parametric two-tailed, t-test (Mann-Whitney) was used for computing statistical significances. *p* value of less than 0.05 were considered significant.

## SUPPLEMENTARY MATERIAL TABLE


