# Supplementary material for: Concordance of anaplastic lymphoma kinase (ALK) gene rearrangements between circulating tumor cells and tumor in non-small cell lung cancer
Source: Oncotarget. 2016 Mar 16;7(17):23251–62. doi: 10.18632/oncotarget.8136 (PMC5029624; doi:10.18632/oncotarget.8136)
Supplement: Supplementary file 1 [file oncotarget-07-23251-s001.pdf]

Concordance of anaplastic lymphoma kinase *(ALK)* gene rearrangements between circulating tumor cells and tumor in non-small cell lung cancer

Supplementary Material

Table S1: Number of circulating tumor cells with *ALK* rearrangement per 1.88 mL of blood

Case no.	Clinical staging	Histological subtype	*ALK* mutation status (Tumor)	Frequency of *ALK* rearranged cells per 1.88 mL of blood
P1	IV	Adenocarcinoma	Positive	15
P2	IV	Adenocarcinoma	Positive	5
P3	IV	Adenocarcinoma	Positive	6
P4	IB	Adenocarcinoma	Positive	11
P5	IV	Adenocarcinoma	Positive	8
P6	IV	Adenocarcinoma	Positive	4
P7	IV	Adenocarcinoma	Positive	4
P8	IV	Adenocarcinoma	Positive	7
P9	IV	NSCLC	Positive	4*
P10	IIIA	Adenocarcinoma	Positive	10*
P11	IV	NSCLC	Positive	4
P12	IV	Adenocarcinoma	Positive	4
P13	IV	Adenocarcinoma	Positive	3
P14	IV	NSCLC	Positive	9
P15	IV	Adenocarcinoma	Negative	0
P16	IV	Adenocarcinoma	Negative	0
P17	IIIB	Adenocarcinoma	Negative	1
P18	IV	Adenocarcinoma	Negative	3
P19	IV	NSCLC	Negative	0
P20	IV	NSCLC	Negative	1^
P21	IV	Adenocarcinoma	Negative	2^
P22	IV	NSCLC	Negative	0
P23	IV	NSCLC	Negative	0
P24	IV	NSCLC	Negative	0
P25	IIIB	NSCLC	Negative	0^
P26	IV	NSCLC	Negative	2
P27	IV	Adenocarcinoma	unknown	6
HD1	Not applicable	Not applicable	Not applicable	2
HD2	Not applicable	Not applicable	Not applicable	2
HD3	Not applicable	Not applicable	Not applicable	0
HD4	Not applicable	Not applicable	Not applicable	2
HD5	Not applicable	Not applicable	Not applicable	2

Abbreviations: *ALK*, anaplastic lymphoma kinase; HD, healthy donor; P, patient; NSCLC, non-small cell lung cancer.

Note: A positive *ALK* mutation is considered when there is a disruption of *ALK* gene resulting in patterns such as 1F1R1G, 1F1R, 2R2G, etc., but excludes the 1F1G pattern and its variants.

*CTC count results based on 7.5 mL blood, not 1.88 mL. These data points have not included determining assay cut-off.

^CTC counts at subsequent time point after initiation of therapy/therapies, not including *ALK* TKI inhibitors.
